# Concerning Mercury (Hg) Levels in the Hair of Children Inhabiting a Volcanically Active Area

**DOI:** 10.3390/toxics13030146

**Published:** 2025-02-21

**Authors:** Rute Fontes, Nádia M. P. Coelho, Patrícia V. Garcia, Filipe Bernardo, Armindo S. Rodrigues

**Affiliations:** 1 FCT, Faculty of Sciences and Technology, University of the Azores, 9501-801 Ponta Delgada, Portugal; rutefontes54@gmail.com (R.F.); patricia.v.garcia@uac.pt (P.V.G.); armindo.s.rodrigues@uac.pt (A.S.R.); 2 cE3c—Centre for Ecology, Evolution and Environmental Changes & CHANGE—Global Change and Sustainability Institute, Azorean Biodiversity Group, University of the Azores, 9501-801 Ponta Delgada, Portugal; 3 IVAR, Institute of Volcanology and Risks Assessment, University of the Azores, 9501-801 Ponta Delgada, Portugal; filipe.mt.bernardo@uac.pt

**Keywords:** volcanism, soil diffuse degassing, gaseous elemental mercury (Hg^0^ or GEM), bioaccumulation, neurotoxicity, neurological disorders

## Abstract

Background: Gaseous elemental mercury (Hg^0^ or GEM) is an atmospheric form of mercury (Hg)—a toxic heavy metal—that is naturally released in volcanic environments. Research with wild mice demonstrates that chronic exposure to a hydrothermal volcanic environment leads to the bioaccumulation of Hg in the lungs, but also in both the central (CNS) and peripheric (PNS) nervous systems, with marked indications of neurotoxicity. Studies addressing human exposure to volcanogenic Hg^0^ are scarce, hence its risks are still unknown. This study aims to evaluate the level of exposure to Hg^0^ in children living in a volcanically active environment. Methodology and main findings: Two groups of school-aged children (from 6 to 9 years old) were part of this study: one with children inhabiting a hydrothermal area (exposed group) and another with children inhabiting an area without volcanic activity (non-exposed group). Hair samples were collected from each individual for Hg level analysis. It was found that the levels of Hg in the hair of exposed children were 4.2 times higher than in that of non-exposed children (≈1797.84 ± 454.92 ppb vs. 430.69 ± 66.43 ppb, respectively). Conclusion: Given the vast health risks Hg poses, the need to monitor the health of populations inhabiting volcanically active areas is highlighted. Because little is known about the fate, modifications, and effects of Hg^0^ in the human body, particularly regarding its effects on the nervous system in children, the development of further research within the scope is strongly encouraged.

## 1. Introduction

Volcanoes are the most prominent sources of natural pollution, being responsible for enriching the environment with hazardous elements. Estimates suggest that more than 14% of the world population lives near active volcanoes, unaware of the silent dangers associated with them [1,2]. Volcanogenic events like ash and gas emissions, soil diffuse degassing, pyroclastic and mud flows, including other phenomena, all contribute to the contamination of soils, water, and atmosphere [3]. Although living in volcanic areas comes with its benefits—like the fertility of soils and geothermal resources–several of the elements commonly associated with these areas are toxic to living organisms, such as arsenic (As), beryllium (Be), cadmium (Cd), mercury (Hg), lead (Pb), and chromium (Cr) [3,4,5,6]. Gaseous elemental mercury (Hg^0^ or GEM), which is an atmospheric form of Hg—a heavy metal that is infamous for its neurotoxicity [7]—is one of the many gases released in volcanic environments, particularly those of hydrothermal origin [8,9,10,11].

Due to its volatility and chemical inertia, as well as the fact that it is the dominant form of both natural and anthropogenic Hg emissions (accounting for >95% of all Hg in the atmosphere), Hg^0^ is a cause for global concern [12,13,14]. In hydrothermal environments, Hg^0^ is mostly released into the atmosphere via soil diffuse degassing [10,11]. The volcanic system of Furnas, located in São Miguel Island, Azores Archipelago, Portugal, comprises an area of hydrothermal volcanism in which Hg^0^ concentrations are below the safety thresholds of 200 ng m^−3^ recommended by the World Health Organization (WHO) [15]. Because of this, Bagnato et al. (2018) [11] proposed that the levels of Hg^0^ released from the Furnas Volcano would not pose a hazard to the populations living in its proximity. However, research with wild mice *Mus musculus* chronically exposed to the hydrothermal volcanic environment of Furnas showed that inhalation is the primary route of exposure and uptake of Hg^0^, leading to the formation of Hg deposits in the lungs [16]. Surprisingly, in other studies with *M. musculus* from Furnas, Hg deposits were also found in blood vessels and the brain, indicating that Hg^0^ can bypass the blood–brain barrier [17]. Further research within the scope demonstrated other alterations regarding *M. musculus*’ central nervous system (CNS), namely, through reactive astrogliosis, astrocyte dysfunction, and neuroinflammation [18,19], but also in their peripheral nervous system (PNS), as seen with a decrease in axon caliber and axonal atrophy along the spinal cord [20]. Taken together, these studies show that, even at environmental doses considered “safe”, chronic exposure to Hg^0^ results in its bioaccumulation in living tissue, paired with a number of adverse effects. Even so, studies addressing human exposure to volcanogenic Hg^0^ are scarce; hence, its risks to populations living in the proximity of volcanoes are still unknown.

While exposure to neurotoxic substances can prove harmful at any age, children are notably more vulnerable to them. This is because their nervous system is at a sensitive stage of development, which renders them at greater risk of developing structural and functional deficits, including when exposures happen at doses much lower than those known to harm adults [21,22,23]. In children, neurological disorders arising from exposure to neurotoxicants can range from cognitive development delay to attention–deficit hyperactivity disorder (ADHD), autism, and neurodegenerative disorders, among others [24,25,26,27].

Several body matrixes, such as blood, saliva, urine, hair, and nails, are often used for human biomonitoring purposes, giving indications about the nutritional status of individuals and exposure to contaminants [28]. Hair is a protein component with a very low metabolic activity that can indicate the overall load of metals in the body. The elemental profile of this matrix is capable of reflecting long-term exposures, as opposed to urine and blood, which are more appropriate for the assessment of short-term exposures [29]. In this sense, hair samples reveal a balance in the body’s mineral content over time, which can only be significantly altered via exposure to or intake of high amounts of trace metals [30].

The aim of this study was to evaluate the level of exposure to Hg^0^ in children living in a volcanically active environment, using Hg levels in hair samples as exposure biomarkers.

## 2. Methodology

### 2.1. Study Sites

**Figure 1:**
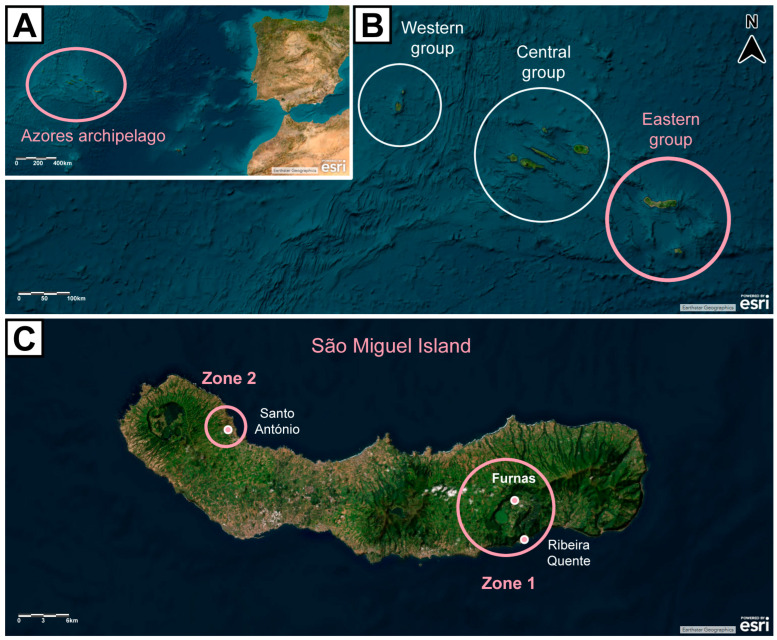
Map of the Azores Archipelago showcasing (**A**) its location in the Atlantic Ocean; (**B**) its geographical groups; (**C**) São Miguel Island and study sites (zone 1 = hydrothermal area, comprising the study group; zone 2 = area without volcanic activity, corresponding to the reference group). Basemap aerial view backgrounds obtained from ESRI ArcGIS online: “World Imagery” [basemap], “World Imagery Map”. Last updated on November 2024. https://www.arcgis.com/home/item.html?id=10df2279f9684e4a9f6a7f08febac2a9 (accessed on 10 December 2024). Attribution to ESRI and other data providers is present in the figure.

The Azores Archipelago, which comprises nine islands belonging to Portugal, is an area with active volcanism located in the Atlantic Ocean, somewhere between 36°45′–39°45′ N and 24°45′–31°17′ W. Its complex tectonic setting—where the Eurasian, African, and American lithospheric plates meet—explains the frequent seismic and volcanic activities recorded [31] (Figure 1A,B). Volcanogenic events in the area are predominantly hydrothermal, with active fumarolic fields, thermal and cold springs rich in carbon dioxide (CO_2_), and zones where soil diffuse degassing occurs [32,33]. São Miguel Island, the largest of the Azores Archipelago, is formed by five volcanic systems, consisting of three active volcanoes (from west to east: Sete Cidades, Fogo, and Furnas), which are linked by two rift zones (Picos and Congro) [31,34,35].

This study was conducted in São Miguel Island, namely, in the Furnas and Ribeira Quente Villages, hydrothermal areas comprising the study group (zone 1), and the Santo António Village, an area without records of volcanic activity, corresponding to the reference group (zone 2) (Figure 1C).

The volcanic activity in the Furnas Village is characterized by secondary volcanism manifestations, such as fumaroles, thermal and cold CO_2_-rich springs, and structures of CO_2_ diffuse degassing. In terms of gases, high amounts of water vapor (H_2_O), CO_2_ (≈1000 t d^−1^) and radioactive radon (Rn^222^) (>10 Bq m^−3^) are released, with traces of hydrogen sulfide (H_2_S), hydrogen (H_2_), nitrogen (N_2_), methane (CH_4_), argon (Ar), helium (He), and carbon monoxide (CO) [33,36]. Aside from these, Hg^0^ is essentially released via fumaroles and soil diffuse degassing, with an output of 9.6 × 10^−5^ t d^−1^ measured in a study area of 0.04 km^2^ inside the Furnas Volcano crater [11].

Along with the Furnas Village, the Ribeira Quente Village was included as integrating “zone 1” (study group) for this study. This is because, as it is located by the southern flank of the Furnas Volcano, there are several areas in this Village with active volcanism manifestations, namely, soil diffuse degassing [37]. In addition, 98% of the houses in this Village were built over the soil diffuse degassing areas [38,39].

In contrast with both Furnas and Ribeira Quente, the Santo António Village does not have volcanic activity and is marked as “zone 2” (reference group) for this study. Although it is geologically represented as the extension of the Sete Cidades volcanic complex, with an area of around 100 km^2^, it does not contain fumarolic nor diffuse degassing sites, with the exception of the Ponta da Ferraria municipality and the beach of Mosteiros; however, the participants from this Village lived about 40 km away from both of these places, far away from such manifestations [40].

### 2.2. Study Groups

Children from the Furnas, Ribeira Quente, and Santo António Villages, with ages between 7 and 9 years old, were included in this study. Participants were eligible based on their age (school-aged) and the place where they lived (i.e., within the study zones). Dietary intake of Hg through fish consumption—which is common in islanders—was not a concern with our study population, given that their diet was more based on meat and dairy products rather than fish (many workers in São Miguel Island are dairy cattle producers).

An informed consent form was signed by each legal representative of the children involved, authorizing their participation in it. The form contained a description of the procedures regarding hair sample collection and other information gathered. Before work began, clarification sessions were held with the children, along with their legal representatives and teachers, about the objectives of this study.

In total, 21 participants were included in this study, which, based on the zone they lived in, were divided into two groups: one with children inhabiting zone 1 (exposed group, *n* = 11) and another with children inhabiting zone 2 (non-exposed group, *n* = 10).

This study was approved by the Ethics Committee of the University of the Azores (REF: 7/2023), verifying that the procedures followed safeguarded the ethical aspects involved in research.

### 2.3. Hair Sampling and Hg Level Analysis

Hair samples were collected from each individual for the determination of Hg levels as biomarkers of exposure to Hg^0^. To eliminate external contamination, each of the hair samples was washed in a sequence of acetone, bi-distilled water, and acetone [41]. After washing, the samples were air-dried at room temperature in a dust-free area. Before analysis, the samples were always kept away from metallic materials and dust to avoid contamination. Next, the samples were cut, homogenized, and weighed into microwave digestion vessels 500 mg ± 10 mg. A combination of nitric acid, hydrogen peroxide, and hydrochloric acid was added as an overnight pretreatment in closed vessels for each batch. The samples were digested and the quantification of Hg (ppb) was achieved via inductively coupled plasma mass spectrometry (ICP-MS) following QOP Hydrogeo Rev. 6.6, carried out in a certified and accredited laboratory (ActLabs, Activation Laboratories Ltd., Canada; ISO 9001:2008 and ISO 17025). Analytical quality control was ensured by method blanks, two triplicate sample digestions, and a spiked sample added to the digestion batch. The method blanks were both below the limit of detection (LOD) for Hg (5 ppb). All human hair samples had Hg concentrations above LOD. Meanwhile, the relative standard deviation (%RSD) values of the two triplicate samples were acceptably low at 1% and 5%. The accuracy of the results was checked by spiking one of the samples in the batch with an internal standard quality control solution containing 10,000 ppb of Hg. The corresponding Hg spike recovery of the analytical method was 91%, which is within the usually accepted range in analytical chemistry (90–110%).

### 2.4. Statistical Analysis

Statistical analysis was carried out in the IBM SPSS Statistics^®^ v. 28.0.1 (142) software. Differences were considered significant when *p* < *0.05*. A Chi-square test was used to compare the distribution of boys and girls between the two groups. The normality of the data was assessed through Q-Q plots. Data regarding age and the levels of Hg (ppb) in children’s hair samples did not present a normal distribution in both groups. Age was compared between groups using a Mann–Whitney *U* test. Similarly, the levels of Hg (ppb) in children’s hair samples were compared using a Mann–Whitney *U* test. Box plots to showcase the differences between groups were made.

## 3. Results and Discussion

Results show no significant differences in age (*U*-test, *p* > *0.05*) or gender distribution (χ^2^ test, *p* > *0.05*) between the two groups (Table 1).

Table 2 shows the results obtained regarding the mean levels of Hg (ppb) in children’s hair samples per group.

The results displayed in Table 2 indicate how much the mean levels of Hg in children’s hair samples significantly differed between groups: they were 4.2 times higher in the hair of exposed children than that of non-exposed children (≈1797.84 ± 454.92 ppb vs. 430.69 ± 66.43 ppb, respectively) (*U*-test, *p* < *0.05*).

**Figure 2:**
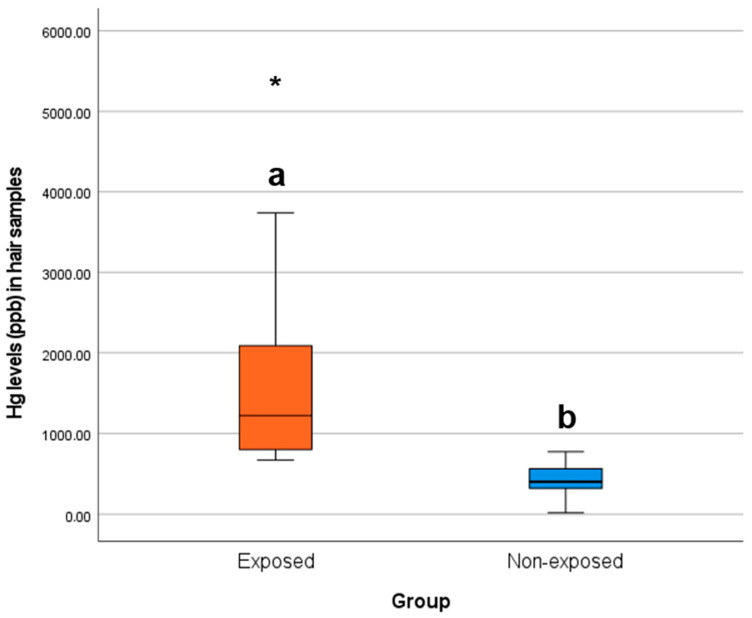
Box plots of the levels of Hg (ppb) in children’s hair samples per group. Different letters (a and b) indicate significant differences (*U*-test, *p* < *0.05*), an asterisk indicates a group outlier, and the line in the middle of each column represents the median of the respective group.

Figure 2 portrays how the data concerning the levels of Hg (ppb) in children’s hair samples per group were distributed.

Based on Figure 2, practically all children in the exposed group presented hair Hg levels above the maximum registered in the non-exposed group (775.89 ppb), which were all significantly (*t*-test, *p* < *0.05*) higher than in the latter.

The toxicity of Hg is well-documented at high-level doses, rendering it infamous for incidents like that of Minamata, Japan, and Iraq [42]. It has been associated with neurological disorders primarily affecting the CNS, such as Alzheimer’s disease and multiple sclerosis, but also the PNS, like Parkinson’s disease [43,44,45]. However, studies addressing low-level exposures to Hg, particularly Hg^0^, along with its fate and effects, are much fewer. Our results clearly show that, although the level exposure to Hg^0^ in the hydrothermal volcanic environment of Furnas is low, chronic exposure leads to the bioaccumulation of Hg in the hair of children, so that its concentration in the hair of exposed individuals was 4.2 times higher than in the hair of non-exposed individuals. Although Hg was indeed present in much higher amounts in the hair of exposed children in comparison to non-exposed, little can be said about the consequences of such exposure because not much is known about the effects of volcanogenic Hg^0^ on the human body. Moreover, when inside the organism, Hg^0^ suffers modifications—a process called chemical speciation—leading to variations in toxicity; hence, the true extent of the hazard it poses to human populations remains unknown [46,47,48,49,50]. In this sense, and knowing, especially, how vulnerable children are to neurotoxicants, the development of further research within the scope is critical.

In a similar study also developed in Furnas, carried out by Amaral et al. (2008) [30], heavy metals like cadmium (Cd), copper (Cu), lead (Pb), rubidium (Rb), and zinc (Zn) were found to be present at higher levels in the hair of men living in the Furnas Village, in comparison to men living in an area without active volcanism. Even though the authors did not evaluate Hg levels in the collected hair samples, it is quite likely that, much like our results showed for children, Hg can equally be found at higher levels in the hair of adults. Later studies in this field of context could include Hg in analyses, as it is a significant toxicant for all age groups.

Some of the available research exploring human low-level Hg exposures highlighting the relationship between hair Hg levels and health outcomes include the works by Yokoo et al. (2003) [51], in Brazil, and Takeuchi et al. (2022) [42], in Japan. Yokoo et al. (2003) [51] conducted the first cross-sectional study addressing human adult exposure to low levels of MeHg, using neuropsychological tests comparable in sensitivity to the methods used in the Faeroes and Seychelles studies. Exposures to Hg in a study population of 129 individuals aged older than 17 were associated with fish consumption, with hair Hg concentration ranging from 0.56 to 13.6 μg/g, with a mean of 4.2 ± 2.4 μg/g (which is around 4000 ppb) and median of 3.7 μg/g. Hair Hg levels were found to be associated with detectable alterations in performance on tests of fine motor speed and dexterity, and concentration, along with the disruption of some aspects of verbal learning and memory—the magnitude of these alterations increased with hair Hg concentration, which was consistent with a dose-dependent effect. From a different perspective, the study of Takeuchi et al. (2022) [42] with young adults assessed the effects of Hg levels on brain morphometry using advanced imaging techniques. In a study population of 920 healthy individuals aged between 18 and 27 years old, exposure to Hg occurred mainly via fish consumption, representing a mean of 2.01 ± 1.15 μg/g in the hair of males and 1.85 ± 1.19 μg/g in the hair of females (around 2000 ppb for both males and females). Such exposure was weakly associated with “(a) lower regional gray matter volume (rGMV) in the left thalamus and left hippocampus, (b) lower regional white matter volume (rWMV) in widespread areas, (c) greater fractional anisotropy (FA) of bilateral white matter tracts, (d) lower mean diffusivity (MD) in widespread areas, particularly bilateral frontal lobe areas and the right basal ganglia, (e) poorer cognitive performance, particularly on tasks requiring cognitive speed, and (f) better mood state (less susceptibility to depressive states)”. Taking into account that exposures to volcanogenic Hg^0^ also tend to happen at low-level doses, in order to shed light on what happens upon exposures to volcanogenic Hg^0^, perhaps a similar methodology to that followed by these authors to reach such conclusions could be applied in future human biomonitoring studies. Furthermore, considering that the concentrations of Hg in the hair of individuals from the study developed by Takeuchi et al. (2022) [42] were similar to those observed in our study (a mean of ≈2000 ppb vs. 1800 ppb, respectively), and bearing in mind that our study involved children—which, as mentioned, are at greater risk due to being in sensitive neurodevelopmental stages—these effects may be even more relevant. Still, it is important to keep in mind that the Hg exposures addressed by Yokoo et al. (2003) [51] and Takeuchi et al. (2022) [42] (mercury vapor from gold mining areas, methylmercury from fish consumption) are different from the ones addressed in our study (volcanogenic Hg^0^); hence, the observed effects when carrying out similar studies may differ.

Ultimately, the results from this study and the former studies discussed support the bioavailability of Hg^0^ in the environment and how it bioaccumulates in living tissue, even at doses considered within a safe range. Still, given our study was performed with island populations, involved very small localities, and depended on voluntary participation, larger and more representative samples of the general population were difficult to obtain. This made it so that our conclusions might not be as robust as they could have been if a larger number of hair samples were included in this study; nonetheless, our conclusions raise very important concerns. With this in mind, and for future studies, it would be indispensable to look for other volcanically active areas to further biomonitor the levels of Hg in children living in these environments. Aside from this, it is worth mentioning that we did not analyze hair samples that were not washed—which could have contributed for a better perception of the amount of Hg actually incorporated into individuals’ hair samples. However, in this case, the Hg values would still be higher if the samples had not been washed. Even so, we recognize that this impossibility of comparing Hg concentrations in samples with and without washing is a limitation of this study. Despite the recognized limitations, the use of hair is a very useful method for monitoring populations chronically exposed to volcanically active environments, since it is not an invasive approach that reflects the degree of exposure of the population.

Another important remark is that inhaled Hg has a half-life of from around 30 to 60 days inside the body, and it is thought to remain in the brain for up to 20 years or more [52,53]. This means that this gas could not only represent a pressing issue for the people living in Furnas, but also for populations living in other areas of the world where Hg^0^ concentrations are deemed below the currently established safety thresholds. On a positive note, studies regarding environmental exposure in recent years show a trend towards a decrease in the acceptable limits for Hg^0^ exposure, reflecting an effort to better safeguard public health [54].

In essence, given that over 14% of the world population lives near active volcanoes, a great number of people could unknowingly be subjected to the risks of Hg^0^ exposure and other volcanogenic hazards. After all, more and more evidence suggests that living chronically exposed to volcanic emissions could be as harmful as living in the proximity of industrial facilities [30].

## 4. Conclusions

Our results showed that the levels of Hg in the hair of children exposed to the hydrothermal volcanic environment of the Furnas Village (São Miguel Island, Azores Archipelago, Portugal) were 4.2 times higher than those of non-exposed children.

Taking into account both the amount of people living in the proximity of active volcanoes (≈14% of the world population) and the known risks that Hg poses, emphasis is put on the need to monitor the health of populations inhabiting volcanically active areas. Moreover, little is still known about the fate, modifications, and effects of Hg^0^ specifically in the human body; hence, the development of further research within this scope is strongly encouraged.

## Figures and Tables

**Table 1:** Descriptive statistics of the age and gender per group of children. * *U*-test, ** χ^2^ test, *p* values do not indicate significant differences (*p* > *0.05*).

Variable	Group	Mean ± Standard Error	Relative Frequency (%)		*p*
Age	Exposed	7.73 ± 0.905	-		*0.099* *
	Non-exposed	8.40 ± 0.699	-		
Gender	Exposed	-	45.5% boys	54.5% girls	*0.119* **
	Non-exposed	-	80% boys	20% girls	

**Table 2:** Descriptive statistics of the levels of Hg (ppb) in children’s hair samples per group. * *U*-test, a *p*-value indicates significant differences (*p* < *0.05*).

Variable	Group	Minimum	Maximum	Mean ± Standard Error	*p*
Hg (ppb) in hair samples	Exposed	672.65	5352.30	1797.84 ± 454.92	***0.001*** *
	Non-exposed	19.05	775.89	430.69 ± 66.43	

## Data Availability

The data that support the findings of this study are available from the corresponding author upon reasonable request.
